# Robotic transversus abdominis release (TAR) for ventral hernia repairs is associated with low surgical site occurrence rates and length of stay despite increasing modifiable comorbidities

**DOI:** 10.1007/s10029-024-03044-6

**Published:** 2024-05-01

**Authors:** A. C. Skoczek, P. W. Ruane, A. B. Holland, J. K. Hamilton, D. L. Fernandez

**Affiliations:** 1 Edward Via College of Osteopathic Medicine – Auburn, 910 S Donahue Dr., Auburn, AL 36830 USA; 2 Edward Via College of Osteopathic Medicine – Carolinas, Spartanburg, SC USA; 3 Lincoln Memorial University DeBusk College of Osteopathic Medicine – Knoxville, Knoxville, TN USA; 4 Crestwood Medical Center, Huntsville, AL USA

**Keywords:** Hernia, Comorbidities, Surgical site infections, Hospital length of stay, Surgical outcomes

## Abstract

**Purpose:**

Modifiable comorbidities (MCMs) have previously been shown to complicate postoperative wound healing occasionally leading to surgeon hesitancy to repair ventral hernias prior to preoperative optimization of comorbidities. This study describes the effects of MCMs on surgical site occurrences (SSOs) and hospital length of stay (LOS) following robotic transversus abdominis release (TAR) with poly-4-hydroxybutyrate (P4HB) resorbable biosynthetic mesh retromuscular sublay for ventral hernia repair in patients who had not undergone preoperative optimization.

**Methods:**

A single-surgeon retrospective review was performed for patients who underwent the robotic TAR procedure with P4HB mesh between January 2015 and May 2022. Patients were stratified by the amount of MCMs present: 0, 1, or 2 + . MCMs included obesity, diabetes, and current tobacco use. Patient data was analyzed for the first 60 days following their operation. Primary outcomes included 60-day SSO rates and hospital LOS.

**Results:**

Three hundred and thirty-four subjects met the inclusion criteria for SSO and prolonged LOS analysis. 16.8% had no MCM, 56.1% had 1 MCM, and 27% had 2 + MCMs. No significant difference in SSO was seen between the 3 groups; however, having 2 + MCMs was significantly associated with increased odds of SSO (odds ratio 3.25, *P* = .019). When the groups were broken down, only having a history of diabetes plus obesity was associated with significantly increased odds of SSO (odds ratio 3.54, *P* = .02). No group showed significantly increased odds of prolonged LOS.

**Conclusion:**

2 + MCMs significantly increase the odds of SSO, specifically in patients who have a history of diabetes and obesity. However, the presence of any number of MCMs was not associated with increased odds of prolonged LOS.

## Introduction

Ventral hernia repair is a highly prevalent surgical intervention, with approximately 600,000 repairs occurring every year [1]. Since 2006, the number of ventral hernia repairs performed each year has nearly doubled, with an estimated annual cost of $9.7 billion [2]. With the high incidence of ventral hernias following gastrointestinal surgery and a growing number of repairs occurring each year, the debate on the most effective surgical technique and biomaterial to close the abdominal wall defect is of high interest [2, 3].

A primary outcome in ventral hernia repair studies is wound morbidity and surgical site occurrences (SSOs) following repair. The presence of modifiable comorbidities (MCMs) that have not been optimized prior to surgical intervention have been shown to increase the odds of short-term complications [3–5]. Specifically, numerous studies have found that diabetes, smoking, and obesity (BMI > 30 kg/m^2^) increase the odds of surgical site infections and regardless of complications, increases the total cost of ventral hernia repairs [3–6]. These MCMs have been shown to complicate the post-operative course following ventral hernia repair; however, the majority of studies have reported these outcomes following an open or laparoscopic approach [3–8]. Research on specific techniques, such as robotic techniques for ventral hernia repair, remains an area where research is limited, particularly regarding long-term outcomes and comparison with traditional methods.

Among the various surgical methods employed, robotic repairs have emerged as a promising technique in addressing complex ventral hernias, both in outcomes and hospital length of stay (LOS) [1, 9–11]. Research on specific biomaterials, such as poly-4-hydroxybutyrate (P4HB) mesh, has shown favorable long- and short-term outcomes, including lower rates of surgical site infections and hernia recurrence, in both open and minimally invasive surgical approaches, with or without component separation [7, 8, 12].

This study aimed to assess the short-term postoperative outcomes in patients who underwent hernia repair using the robotic transversus abdominis release (TAR) method with P4HB resorbable biosynthetic mesh retromuscular sublay. The integration of robotic assistance and advanced biomaterials, such as P4HB mesh, in ventral hernia repair holds promise in minimizing recurrence rates and postoperative complications [7, 8, 11–14]. However, the interaction between these advancements and modifiable patient factors remains [2, 5, 6, 15]. Due to the limited research on this technique and concerns regarding MCMs, this study aims to contribute to refining surgical strategies and enhancing the overall quality of care for patients undergoing ventral hernia repair.

## Materials and methods

### Study design

This study represents a retrospective review of prospectively managed electronic health records to assess the short-term outcomes of the robotic TAR technique with P4HB resorbable biosynthetic mesh retromuscular sublay. This study was reviewed by the Edward Via College of Osteopathic Medicine institutional review board and was granted exemption status. All patients who had undergone a ventral hernia repair utilizing the aforementioned technique performed by a single surgeon were identified between January 2015 and May 2022. The primary aim of this study was to evaluate the short-term outcomes in patients considered at risk for post-operative complications due to their MCMs. Only patients who had undergone elective hernia repair and completed their appropriate follow-up were included. Follow-up included a 2-week post-operative appointment and a 6-week post-operative appointment. Due to inconsistency of future follow up appointments, patient data was only included for the first 60-days postoperatively. Patients were excluded if they had undergone an emergent hernia repair or a revision from a previous robotic TAR procedure and patients with incomplete medical records. In addition, patients who underwent a contaminated procedure were excluded.

MCMs were defined as a comorbidity that has the potential to be optimized before an elective hernia repair [16]. In accordance with the modified VHWG grading scale, patients with obesity, diabetes, and active smokers are at risk for increased rates of wound events postoperatively. Individuals with a body mass index (BMI) of ≥ 30 kg/m^2^ were defined as obese based on the Centers for Disease Control and Prevention guidelines [15]. The study population was stratified based on the number of MCMs they possessed: 0, 1, or 2 + MCMs. To be included in the 0 MCM group, patients had to be not obese with a BMI < 30 kg/m^2^, non-diabetics, and not actively smoking. Patients in the 1 MCM group only had one comorbidity, either a BMI of > 30 kg/m^2^, diabetes, or was an active smoker. Patients in the 2 + MCM group had a combination of any two or more MCMs—for example, being obese with diabetes or obese with diabetes and being an active smoker.

In addition to the primary analysis of the effects of MCMs, a secondary analysis was conducted to evaluate the relationship between different BMI classes and SSOs. For the secondary analysis, patients were grouped based on BMI as either healthy (BMI ≥ 18.5 and < 25 kg/m^2^), overweight (BMI ≥ 25 and < 30 kg/m^2^), obese (BMI ≥ 30 and < 40 kg/m^2^), and morbidly obese (BMI ≥ 40 kg/m^2^). Due to the short follow up period in this study, recurrence rates were not studied but have been previously published [11].

### Surgical technique

All patients underwent robotic TAR with P4HB resorbable biosynthetic mesh sublay. All included cases were clean with no contamination. Following induction of general anesthesia, entry into the abdomen, and docking of the da Vinci^®^ robotic surgical system, bilateral rectus muscle flaps were created. To create the muscle flap, the posterior rectus sheath was divided along the medial aspect of the right and left rectus sheath. The dissection began medially and was taken out to the semilunar line. Following the dissection, the transversalis fascia, and the transversus abdominis muscle were divided, releasing the rectus sheath medially. The dissection was taken out further laterally, releasing the transversus abdominis muscle from the posterior rectus sheath. This dissection was continued out to the retroperitoneum.

The anterior fascial defect was repaired using a barbed self-locking suture, imbricating the hernia sac and reapproximating the fascial edges. P4HB mesh was selected due to surgeon discretion. Non-resorbable biosynthetic meshes were not used. P4HB mesh size was estimated by externally measuring the fascial defect and secured to the abdominal wall as a retromuscular sublay using four tacking sutures, one in each corner. An intraabdominal drain was not placed in any of the included patients. The surgical technique performed was explained in greater detail in a prior study by Skoczek, et al. [11]. The intraoperative technique was identical for all participants included.

### Statistical analysis

The two primary outcomes analyzed in this study included the 60-day SSO rate and hospital LOS following the procedure. Data was summarized with descriptive statistics using numbers and percentages for categorical variables and means, standard deviations, medians, and interquartile ranges (IRQs) for continuous variables where appropriate. Pearson chi-squared test for categorical variables, Fischer exact test for zero counts, and the Kruskal–Wallis test or analysis of variance for continuous variables were conducted to compare the patient groups, individual MCMs, and combinations of MCMs. Analysis was conducted using R: The Project for Statistical Computing [17].

## Results

### Study participant demographics and comorbidities

A total of 334 patients met the inclusion criteria and underwent the robotic TAR with P4HB mesh between January 2015 and May 2022. Of these, 17% (*n* = 58) had no MCM, 56% (*n* = 193) had 1 MCM, and 27% (*n* = 93) had 2 + MCMs. Patient demographics, including age, sex, number of previous abdominal surgeries, hernia diameter, hernia area, and BMI, are presented in Table 1. The median age of the population was 58 years old (Q1–Q3, 46–67 years), with no significant difference seen between groups (*P* = .47). In addition, no significant difference between sex or number of previous abdominal surgeries was observed (*P* = .13 and *P* = .17 respectively).

**Table 1:** Demographics

Characteristic	*N*	No MCM	1 MCM	2 + MCM	Combined	Test statistic
		*N* = 58	*N* = 193	*N* = 93	*N* = 344	
Age (median [Q1–Q3])	344	60 [44–70]	58 [47–68]	57 [45–63]	58 [46–67]	*P* = 0.47^ʄ^
Advanced Age (> 70 years)	344	26% (15)	18% (34)	9.7% (9)	17% (58)	*P* = 0.032^ɸ^
Sex	344					*P* = 0.13^ɸ^
Female		78% (45)	63% (122)	67% (62)	67% (229)	
Male		22% (13)	37% (71)	33% (31)	33% (115)	
Number of previous abdominal surgeries	344					*P* = 0.17^ɸ^
0		17% (10)	17% (32)	9.7% (9)	15% (51)	
1		17% (10)	21% (41)	16% (15)	19% (66)	
2		26% (15)	21% (41)	31% (29)	25% (85)	
3		8.6% (5)	15% (29)	22% (20)	16% (54)	
4		16% (9)	8.3% (16)	11% (10)	10% (35)	
5 +		16% (9)	18% (34)	11% (10)	15% (53)	
Hernia diameter in cm (mean ± SD)	344	11.8 ± 4.5	12.4 ± 5.0	13.7 ± 5.8	12.7 ± 5.2	*P* = 0.09^ǂ^
Hernia area in cm (mean ± SD)	344	126 ± 105	141 ± 125	174 ± 158	147 ± 133	*P* = 0.10^ǂ^
BMI (mean ± SD)	344	26 ± 3	35 ± 7	38 ± 7	34 ± 7	*P* < 0.001ǂ

^ʄ^Kruskal–Wallis test

^ɸ^Pearson test

^ǂ^Analysis of variance test

Amongst the patients, the mean hernia diameter was 12.7 ± 5.2 cm, with a mean hernia area of 147 ± 133 cm. No difference was observed between the three groups, with a mean hernia diameter of 11.8 ± 4.5 cm in no MCM group, 12.4 ± 5.0 cm in 1 MCM group, and 13.7 ± 5.8 cm in 2 + MCMs group (*P* = .09). The mean BMI of the population was 34 ± 7 kg/m^2^, with 2 + MCMs having the highest mean BMI among the three groups (26 ± 3 kg/m^2^ in the no MCM group, 35 ± 7 kg/m^2^ in the 1 MCM group, and 38 ± 7 kg/m^2^ in the 2 + MCMs group; *P* < .001).

The distribution of comorbidities between the 3 groups can be seen in Table 2. Obesity was the most common MCM present, with 80% (*n* = 155) of the 1 MCM group having a BMI of ≥ 30 kg/m^2^. 10% (*n* = 20) of the 1 MCM group were diabetic, and 9.3% (*n* = 18) were active smokers. In patients with 2 + MCMs, 96% (*n* = 89) were obese, 66% (*n* = 61) were diabetic, and 51% (*n* = 47) were active smokers at the time of surgery. Patients with a previous diagnosis of diabetes were included irrespective of their HbA1c level prior to surgery or if they were currently compliant with their diabetes treatment.

**Table 2:** Distribution of comorbidities between groups

	1 MCM	2 + MCM
	*N* = 193	*N* = 93
Obesity	80% (155)	96% (89)
Diabetes	10% (20)	66% (61)
Smoking	9.3% (18)	51% (47)
BMI (mean ± SD)	35 ± 7	38 ± 7

### SSO within 60 days by MCM

Of the 344 patients in the cohort, 15 (4.4%) experienced a superficial SSO within the first 60 days following surgery. This included 1 patient in the no MCM group (1.7%), 6 patients in the 1 MCM group (3.1%), and 8 patients in the 2 + MCM group (*P* = .087). Of the SSOs experienced, 10 of the 15 (66.7%) were superficial abscesses, 1 (6.6%) was cellulitis around the wound, and 4 (26.6%) were the formation of a seroma. While no significant difference in SSO was observed between groups (*P* = .087), a significant difference in abscess formation was observed (0% [*n* = 0] in the no MCM group, 2.1% [*n* = 4] in the 1 MCM group, and 6.5% [*n* = 6] in the 2 + MCMs group; *P* = .05).

On multivariable logistic regression, patients in the no MCM group and 1 MCM group had no significantly increased odds of a SSO (OR = .386, 95% CI [.016–1.99] and OR = .511, 95% CI [.165–1.48} respectively). Patients with 2 + MCMs, however, did show an increased odds of SSO within 60 days (OR = 3.25, 95% CI [1.12–9.74]; *P* = .019). Table 3 presents a summary of these analyses.

**Table 3:** SSO analysis – MCM

	No MCM	1 MCM	2 + MCM	Combined	*P* value
	*N* = 58	*N* = 193	*N* = 93	*N* = 344	
*Univariate analysis*					
SSO	1.7% (1)	3.1% (6)	8.6% (8)	4.4% (15)	0.087
Seroma	0% (0)	1.0% (2)	2.2% (2)	1.2% (4)	0.6
Cellulitis	1.7% (1)	0% (0)	0% (0)	0.3% (1)	0.2
Superficial abscess	0% (0)	2.1% (4)	6.5% (6)	2.9% (10)	**0.05**

Variable	Odds ratio	CI 95%	*P* value
*Multivariable logistic regression*			
No MCM	0.386	0.016–1.99	0.28
1 MCM	0.511	0.165–1.48	0.20
2 + MCM	3.25	1.12–9.74	**0.019**

Bold values indicate statistical significance (P < 0.05)

For further analysis, each of the 2 comorbid groups (1 MCM and 2 + MCMs) were then broken down to include all possible comorbidities and their combinations. No significant difference in SSO rates was seen between the different comorbidity types and comorbidity combinations (*P* = .11). On multivariable logistic regression, having the combination of diabetes plus obesity was the only comorbidity or combination of comorbidities to show an increased odds of SSOs (OR = 5.16, 95% CI [1.75–14.43]; *P* = .0006). Of note, having a singular MCM, either diabetes or obesity, did not show an increased odds of SSO (OR = 1.14, 95% CI [.046–6.14] and OR = .257, 95% CI [.056–.817] respectively). Table 4 summarizes the above findings. Due to the retrospective nature of the study, data on SSOs requiring procedural intervention and/or hospital readmission were not recorded and analyzed.

**Table 4:** Analysis of SSO on comorbidity breakdown

	No comorbidity	Diabetes	Obesity	Smoking	Diabetes + smoking	Diabetes + obesity	Obesity + smoking	Diabetes + obesity + smoking	Combined	*P* value
	*N* = 58	*N* = 20	*N* = 155	*N* = 18	*N* = 4	*N* = 4*6*	*N* = 32	*N* = 11	*N* = 344	
*Univariate analysis*										
SSO within 60 days	1.7% (1)	5% (1)	3.2% (5)	0% (0)	0% (0)	11% (5)	3.1% (1)	18% (2)	4.4% (15)	0.11

Variable	Odds Ratio	95% CI	*P* value
*Multivariable logistic regression SSO*			
No comorbidity	0.386	0.016–1.99	0.31
Diabetes	1.31	0.052–7.16	0.81
Obesity	0.61	0.181–1.77	0.39
Diabetes + obesity	3.54	1.03–10.68	0.02
Obesity + smoking	0.77	0.031–4.1	0.71
Diabetes + obesity + smoking	5.68	0.733–25.74	0.087

### SSO within 60 days by obesity class

As a secondary analysis, obese patients were stratified based on their BMI classification; underweight, healthy, overweight, obese, and morbidly obese. A total of 0.3% (*n* = 1) of patients had an underweight BMI, 7.8% (*n* = 26) had a healthy BMI, 21.2% (*n* = 72) had an overweight BMI, 53% (*n* = 177) were obese, and 19.5% (*n* = 67) were morbidly obese. No significant differences in age, sex, or number of previous surgeries were observed between groups. SSOs occurred in 0% of the underweight patients, 3.8% (*n* = 1) of the healthy BMI patients, 1.4% (*n* = 1) of the overweight patients, 5.1% (*n* = 9) of the obese patients, and 6% (*n* = 4) of the morbidly obese patients. No significant difference was seen between groups (*P* = .68). In addition, no difference in rates of seromas, cellulitis, or abscesses was seen between groups (*P* = .43, *P* = .44, *P* = .34 respectively).

On multivariable logistic regression none of the BMI groups showed an increased odds of SSO within 60 days; healthy (OR = .979, 95% CI [.039–5.24]), overweight (OR = .289, 95% CI [.012–1.48]), obese (OR = 1.42, 95% CI [.49–4.42]), and morbidly obese (OR = 1.57, 95% CI [.41–4.84]). Results of the obesity class analysis can be seen in Table 5.

**Table 5:** Surgical site occurrence analysis – obesity class

	Underweight	Healthy	Overweight	Obese	Morbid obesity	Combined	*P* value
	*N* = 1	*N* = 26	*N* = 73	*N* = 177	*N* = 67	*N* = 344	
*Univariate analysis*							
SSO	0% (0)	3.8% (1)	1.4% (1)	5.1% (9)	6.0% (4)	4.4% (15)	0.68
Seroma	0% (0)	0% (0)	0% (0)	2.3% (4)	0% (0)	1.2% (4)	0.43
Cellulitis	0% (0)	0% (0)	1.4% (1)	0% (0)	0% (0)	0.3% (1)	0.44
Superficial abscess	0% (0)	3.8% (1)	0% (0)	2.8% (5)	6.0% (4)	2.9% (10)	0.34

Variable	Odds ratio	CI 95%	*P* value
*Multivariable logistic regression*			
Healthy	0.979	0.039–5.24	0.89
Overweight	0.289	0.012–1.48	0.21
Obese	1.42	0.49–4.42	0.49
Morbid obesity	1.57	0.41–4.84	0.48

### LOS by MCM

Of the 344 participants, patients stayed in the hospital for 1.54 ± 1.85 days. A significant difference was seen between the 3 groups (no MCM, 1 MCM, and 2 + MCMs), with the no MCM group having an average hospital stay of 1.74 ± 1.65 days, the 1 MCM group having an average LOS of 1.31 ± 1.20 days, and the 2 + MCM group having an average LOS of 1.91 ± 1.85 (*P* = .027). In addition, 8.6% (*n* = 5) patients in the no MCM group, 2.1% (*n* = 4) patients in the 1 MCM group, and 8.6% (*n* = 8) of patients in the 2 + MCM group had a prolonged hospital LOS of greater than four days with a significant difference observed (*P* = .014).

While a difference of prolonged LOS of greater than four days was observed between groups, no group showed an increased odds of having a prolonged LOS: 0 MCM (OR = 2.18, 95% CI [.66–6.24], 1 MCM (OR = .23, 95% CI [.062–.68], and 2 + MCMs (OR = 2.53, 95% CI [.91–6.9]) (Table 6). On comorbidity breakdown, a significant difference was seen between groups (*P* = 0.034) however, no group had an increased odds of prolonged LOS (Table 7).

**Table 6:** LOS analysis – MCM

	No MCM	1 MCM	2 + MCM	Combined	*P* value
	*N* = 58	*N* = 193	*N* = 93	*N* = 344	
*Univariate analysis*					
LOS (mean ± SD)	1.74 ± 1.65	1.31 ± 1.20	1.91 ± 1.85	1.54 ± 1.85	**0.027**
LOS > 4 days	8.6% (5)	2.1% (4)	8.6% (8)	4.9% (17)	**0.014**

Variable	Odds ratio	CI 95%	*P* value
*Multivariable logistic regression*			
No MCM	2.18	0.66–6.24	0.156
1 MCM	0.23	0.062–0.68	**0.006**
2 + MCM	2.53	0.91–6.9	0.057

Bold values indicate statistical significance (P < 0.05)

## Discussion

In recent years, there has been an increase in minimally invasive surgeries due to the rise in the robotic surgical approach. However, limited research exists on how robotic surgery impacts short-term outcomes in ventral hernia repairs. This research focus was to investigate the effect of MCMs on the short-term outcomes of robotic TAR with P4HB mesh for ventral hernia repair in patients who did not undergo preoperative optimization. Our results showed favorable outcomes in patients with MCMs, including SSOs and hospital LOS. However, an increased odds of surgical site occurrences was noted in patients with diabetes plus obesity.

Prior research has highlighted the influence of MCMs, particularly obesity and diabetes, on short-term outcomes like SSOs [3, 6, 18–20]. A review published by Pierpont et al. in 2018 noted that obesity was associated with higher rates of postoperative complications like wound and adipose healing in general surgical procedures due to decreased vascularity at the wound site, cellular and molecular alterations in inflammatory cell lines, and increased oxidative stress. They noted that diabetes-related wound morbidity can be decreased by 30% if patients undergo a 10-kg weight loss prior to surgery; however, patients with large complex ventral hernias often struggle with weight loss due to their inability to exercise secondary to their hernia [20]. Similarly, a meta-analysis of 90 studies, including multiple procedure types by Martin et al. in 2015, found that diabetes increased surgical site infection risk by 53% [19]. Park et al. in [18] found that patients undergoing elective non-emergent open ventral hernia repair with mesh stepwise increase in surgical site infection rate as BMI increased above 24.2 kg/m^2^, with the highest risk being in smokers with BMI > 42.3 kg/m^2^. While these studies have shown increased short-term outcome risks in patients with MCMs, there is a gap in understanding how the confluence of multiple comorbidities affects outcomes in the context of advanced minimally invasive surgical procedures like the robotic TAR.

Newer research analyzing minimally invasive techniques has found robotic surgery to be associated with reduced rates of SSOs compared to open surgery. Kudsi et al. [21] noted that robotic ventral hernia repair resulted in surgical site event rates of 12.2%, which was 9.3% less than open ventral hernia repair surgical site event rates. In addition, a recent study by Gaskins et al. [22] found that the robotic technique had no difference in SSO rates compared to an open approach in patients with a BMI above 35 kg/m^2^ and who were either current smokers or diabetics. Specifically related to the TAR approach, a 2021 study by Bracale et al. found that robotic TAR was associated with a lower risk of SSOs compared to open TAR (5.3% vs 11.5%), though there was no significant difference found when evaluating SSOs that required intervention, and the comorbidities evaluated in the study (gender, BMI, and diabetes) did not impact complication risks [23]. While limited, this newer research on robotic techniques is beginning to demonstrate that minimally invasive robotic techniques may be a viable option for ventral hernia surgical repair in complex patients. However, the research on specific techniques and their outcomes is sparse. Our study addresses this gap, suggesting that the robotic TAR technique successfully decreases the burden of multiple comorbidities on surgical outcomes.

In this study, the incidence of SSOs within 60 days post-surgery was 4.4% in all groups, significantly lower than other published open and robotic techniques; however, similar to other published studies on the robotic TAR technique [24]. A notable increase in abscess formation was seen in the group with two or more MCMs. Patients with two or more comorbidities experienced rates of SSOs at 8.6%, which, while higher than in patients with no MCMs (1.7%), is still lower than what has been shown in other studies of other techniques. Moreover, the overall SSO rates did not significantly differ between groups. When single comorbidities and a combination of comorbidities were analyzed, the combination of diabetes plus obesity notably increased the odds of SSOs (OR = 3.54). Having either condition alone, however, did not significantly increase the odds. Furthermore, while obesity alone did not increase the odds of SSOs (OR = .61), a secondary analysis of different BMI classes was conducted. Our data shows that even in patients with a BMI of > 40 kg/m^2^, SSOs occurred at a rate as low as 6%, with no increased odds of SSOs in morbidly obese patients.

While SSOs constitute a significant burden following hernia repair, hospital length of stay significantly increases the cost burden of ventral hernia repairs. In 2006, a ventral hernia repair with a hospital length of stay cost between $15,394 and $16,404, with an increase of approximately $1000 yearly from 2001 [25]. Compared to open repairs, robotic ventral hernia repairs cost approximately $2000 more per surgery in the inpatient setting, though this cost is offset by the higher post-discharge expenses incurred in open repair patients. Overall, this results in comparable total costs compared to open repairs [21]. With the high cost of inpatient ventral hernia repairs and the increased cost of robotic repairs, decreasing hospital length of stay is vital to decreasing overall robotic repair costs and enhancing its utility compared to an open approach.

Previous studies have shown that an extended LOS was observed in patients with comorbid conditions undergoing various open surgical procedures [26]. While comorbid conditions were not factored into their analysis, a study by Carbonell et al. [27] found that robotic hernia repairs resulted in 1 less day of hospital admission, with a significant difference in hospital LOS between robotic and open repairs. No studies have analyzed the effects of MCMs on hospital LOS in robotic component separation repairs. Our findings did show a significant difference in patients with MCMs, with patients with no MCMs having an average LOS of 1.71 days and patients with two or more MCMs having an average LOS of 1.94 days. However, no group showed significantly increased odds of prolonged hospital stay exceeding four days (Table 7).

**Table 7:** Analysis of LOS on comorbidity breakdown

	No comorbidity	Diabetes	Obesity	Smoking	Diabetes + smoking	Diabetes + obesity	Obesity + smoking	Diabetes + obesity + smoking	Combined	*P* value
	*N* = 58	*N* = 20	*N* = 155	*N* = 18	*N* = 4	*N* = 4*6*	*N* = 32	*N* = 11	*N* = 344	
*Univariate analysis*										
LOS (mean ± SD)	1.74 ± 1.65	1.70 ± 1.45	1.26 ± 1.18	1.28 ± 1.02	3.50 ± 2.38	2.26 ± 2.21	1.38 ± 1.18	1.45 ± .93	1.54 ± 1.50	**0.034**
LOS > 4 days	8.6% (5)	5% (1)	1.9% (3)	0% (0)	25% (1)	15% (7)	0% (0)	0% (0)	4.9% (17)	**0.005**

Variable	Odds Ratio	95% CI	*P* value
*Multivariable logistic regression LOS* > *4 days*			
No comorbidity	2.18	0.66–6.24	0.156
Diabetes	1.14	0.0456–6.14	0.99
Obesity	0.257	0.056–.817	**0.02**
Diabetes + smoking	7.22	0.24–66.12	0.197
Diabetes + obesity	5.16	1.75–14.43	0.0006

Bold values indicate statistical significance (P < 0.05)

Our study's uniqueness lies in its evaluation of the robotic TAR technique with P4HB mesh, a combination not extensively explored in previous research, particularly in a cohort with a high prevalence of comorbidities. Nevertheless, this study is not without limitations. Its single-surgeon, single-technique nature, coupled with a small sample size, may limit the generalizability of the findings. The relatively small sample size resulted in a low statistical power. Therefore, while our study did not find significant differences between groups for SSOs, it cannot be concluded that there is no difference. Additionally, this study only reported SSO rates and hospital LOS for patients undergoing robotic procedures, which limits the comparison between different surgical approaches. Future research should involve larger, multicenter studies to validate these results and explore the explore the efficacy of preoperative interventions targeting MCMs.

## Conclusion

Our research demonstrated positive short-term postoperative outcomes in patients with MCMs whose MCMs were not optimized preoperatively, including no increased odds of SSOs in patients with a singular MCM or prolonged LOS in patients with any number of MCMs. While the robotic TAR technique with P4HB mesh represents a significant advancement in ventral hernia repair, the presence of multiple MCMs, particularly diabetes plus obesity, remains a risk factor for increased SSOs. Further studies are required to increase generalizability and directly compare its efficacy to other currently available approaches.
